# Clinical Features Differ Between Patients With Vertigo Attack Only and Weakness Attack Accompanying Vertigo Before Vertebrobasilar Stroke: A Retrospective Study

**DOI:** 10.3389/fneur.2022.928902

**Published:** 2022-07-27

**Authors:** Yalan Deng, Lei Zhang, Rongsen Zhang, Jingfeng Duan, Jiabing Huang, Dongxu Qiu

**Affiliations:** ^1^Department of Oncology, NHC Key Laboratory of Cancer Proteomics, Laboratory of Structural Biology, National Clinical Research Center for Geriatric Disorders, Xiangya Hospital, Central South University, Changsha, China; ^2^Department of Neurology, Xiangya Hospital, Central South University, Changsha, China; ^3^Department of Ultrasonography, Second Xiangya Hospital, Central South University, Changsha, China; ^4^Department of Neurology, Third Hospital of Changsha, Changsha, China; ^5^Department of Cardiology, The Second Affiliated Hospital of Nanchang University, Nanchang, China

**Keywords:** vertigo attack, cerebral infarction, weakness attack, vertebrobasilar stroke, infarction

## Abstract

**Objective:**

To determine the different clinical features of patients with vertigo attacks alone and of those with weakness accompanying vertigo attacks before the vertebrobasilar ischemic stroke.

**Methods:**

In this 4-year retrospective study, we manually screened the medical records of 209 patients, hospitalized with vertigo attack as the main complaint who were finally diagnosed with acute vertebrobasilar ischemic stroke. Patients were divided into two groups according to their symptoms: patients who only experienced vertigo attacks prior to the vertebrobasilar stroke (VO group) and patients who had both vertigo and weakness attacks (VW group) prior to the stroke. Clinical parameters, such as infarction site and volume, relative risk factors, ABCD^2^ score, and medical intervention, were compared between the two groups.

**Results:**

The prevalence of hypertension was higher in the Vertigo attacks only (VO) group (42.2 vs. 29.0%, *p* < 0.05). The total cerebral infarction volume in the VO group was larger than the Vertigo and weakness attacks (VW) group (4.44 vs. 2.12 cm^3^, *p* < 0.05). Additionally, the cerebellum was more likely to be affected in the VO group. In contrast, patients in the VW group had higher carotid stenosis (14.2 vs. 27.2%, p < 0.05) and ABCD^2^ score (2.1 ± 1.2 vs. 3.6 ± 1.5, *p* = 0.02). The percentage of patients with medullary infarctions also increased in the VW group. Vertigo attack events occurred more frequently in the VW group (median 2.4 vs. 4.3, *p* < 0.04). We also found that the patients in the VW group were more likely to seek medical intervention after vertigo.

**Conclusions:**

Clinical parameters, such as infarction location, relative risk factors, and ABCD^2^ score, differed between patients with vertigo symptoms with or without weakness attacks. These findings highlight the different clinical features of patients with vertigo attack only and those with weakness attacks accompanying vertigo prior to vertebrobasilar ischemic stroke.

## Introduction

Currently, recurrent vertigo attacks, especially for cerebrovascular disorders, are gaining increased attention. Vertigo attacks presenting with normal neurological examination results are common among patients with vertebrobasilar stroke. However, ascribing transient vertigo to ischemic events remains a diagnostic challenge due to the lack of highly sensitive diagnostic instruments (1, 2). Since recurrent vertigo can be the only symptom of posterior circulation ischemia, isolated vertigo symptoms are always diagnosed as Ménierè's disease or migraine rather than transient ischemic attack (3–6). Moreover, the characteristics of isolated vertigo attacks in vertebrobasilar transient ischemic attacks are highly variable and atypical compared to other neurological deficits. These accompanying neurological deficits were observed in 11% of patients with vertebrobasilar ischemic stroke (7, 8). As stroke is the leading cause of severe disability, failure to recognize focal deficits prior to vertebrobasilar stroke may result in worse outcomes (9, 10). Thus, highlighting the differences in patients with vertigo attacks only and vertigo accompanying weakness attacks is important for secondary stroke prevention.

Vertigo symptoms are commonly present in 47–75% of patients due to posterior circulation hypoperfusion (11, 12). Recurrent vertigo from vertebrobasilar infarction is increasingly being recognized. However, in some cases, more than one focal neurological deficit can be observed prior to vertebrobasilar stroke (13). For example, patients may experience sudden weakness in the face, arm, leg, or soft palate if hypoperfusion is afflicted in the medulla (14, 15). Therefore, neurological deficits may transition from one territory to another over time because the territory is affected by hypoperfusion. Therefore, it is necessary to clarify the clinical characteristics of vertigo attacks with and without vertigo and weakness prior to the vertebrobasilar stroke. However, no study has been conducted to determine the differences between the two. We conducted a retrospective analysis by enrolling patients with vertigo with or without a weakness attack prior to the vertebrobasilar stroke. This study aimed to determine the clinical parameters, such as infarction volume and location, and relative risk factors, in patients with vertigo symptoms only and vertigo accompanying weakness attacks preceding vertebrobasilar stroke.

## Materials and Methods

### Study Subjects

This prospective study included two groups of patients with suspected vascular-associated vertigo. All patients involved in this study had vertigo symptoms, with or without a history of weakness. These patients were hospitalized at the Second Xiangya Hospital, Xiangya Hospital, and the Third Hospital of Changsha. All included patients were diagnosed with acute vertebrobasilar ischemic stroke. The study was approved by the ethics committee of Xiangya Hospital. All the patients included in this study signed the consent form.

### Definition of Vertebrobasilar Ischemic Stroke

All patients received targeted enquiries from a trained neurologist. The main items were as follows: whether they had a weakness attack on the face, arm, leg, or soft palate within 3 months prior to the acute stroke; date of symptom onset; baseline characteristics; duration and type of symptoms; and time of first seeking medical intervention. Similar questions were asked regarding the history of vertigo attacks. The Bárány Society criteria were introduced to define vertigo attacks for this study (16–18). If they answered affirmatively, related questions, such as a detailed description of the vertigo attack, the exact time of onset, the frequency and duration of symptoms, and whether they received medical therapy, were also asked. Vertebrobasilar ischemic stroke was determined as follows: 1) acute focal neurological deficits lasting for more than 24 h, 2) magnetic resonance imaging (MRI) supported the ischemic infarction corresponding to the neurological deficits, and 3) the final diagnosis of vertebrobasilar ischemic stroke was confirmed by two different trained neurologists based on the computed tomography findings, MRI evidence, and clinical presentations.

### Evaluation of the Vascular Risk Factors

Hypertension was determined based on the following criteria: the previous diagnosis of hypertension, diastolic blood pressure >90 mm Hg, and/or systolic blood pressure > 140 mm Hg. Diabetes mellitus was defined as a history of relative disease or diagnostic criteria, according to the diabetes mellitus standard. Other vascular risk factors included alcohol consumption (100 ml of wine or 300 ml of beer per day, lasting for the past 3 month), hyperlipidemia (cholesterol >5.17 mmol/L and/or triglycerides >1.71 mmol/L), and smoking (≥1 cigarette per day; smoking history > 9 months). Trained neurologists performed carotid artery ultrasonography. Artery stenosis >50% was defined as the diagnostic criterion for extracranial arterial stenosis (15).

### Determination of the Infarction Focal Volume

GE Signa HDX 3.0T MRI (Fairfield, USA) was used to capture MRI scans. The apparent diffusion coefficient, T1- and T2-weighted scans, fluid-attenuated inversion recovery imaging, and diffusion-weighted imaging data were collected for each patient. A field of view of 220 mm × 220 mm, slice thickness of 5.0 mm, and maximum-intensity projection of a three-dimensional volume were performed for imaging reconstruction and raw data collection. All detection procedures were conducted using a post-processing GE machine (Siemens, Inc., Munich, Germany). Two experienced observers were independently appointed to reduce the unconscious bias involved in the data analysis.

### Statistical Analysis

The SPSS software (version 23.0; IBM, Chicago, IL, USA) was used to perform the statistical analyses. The two independent group comparisons were analyzed by unpaired two-sample *t*-test, and comparisons between three or more groups were performed using one-way analysis of variance; mean ± standard deviation was used to express the continuous variables with normal distribution; *p* < 0.05 was considered statistically significant in all tests.

## Results

**Figure 1:**
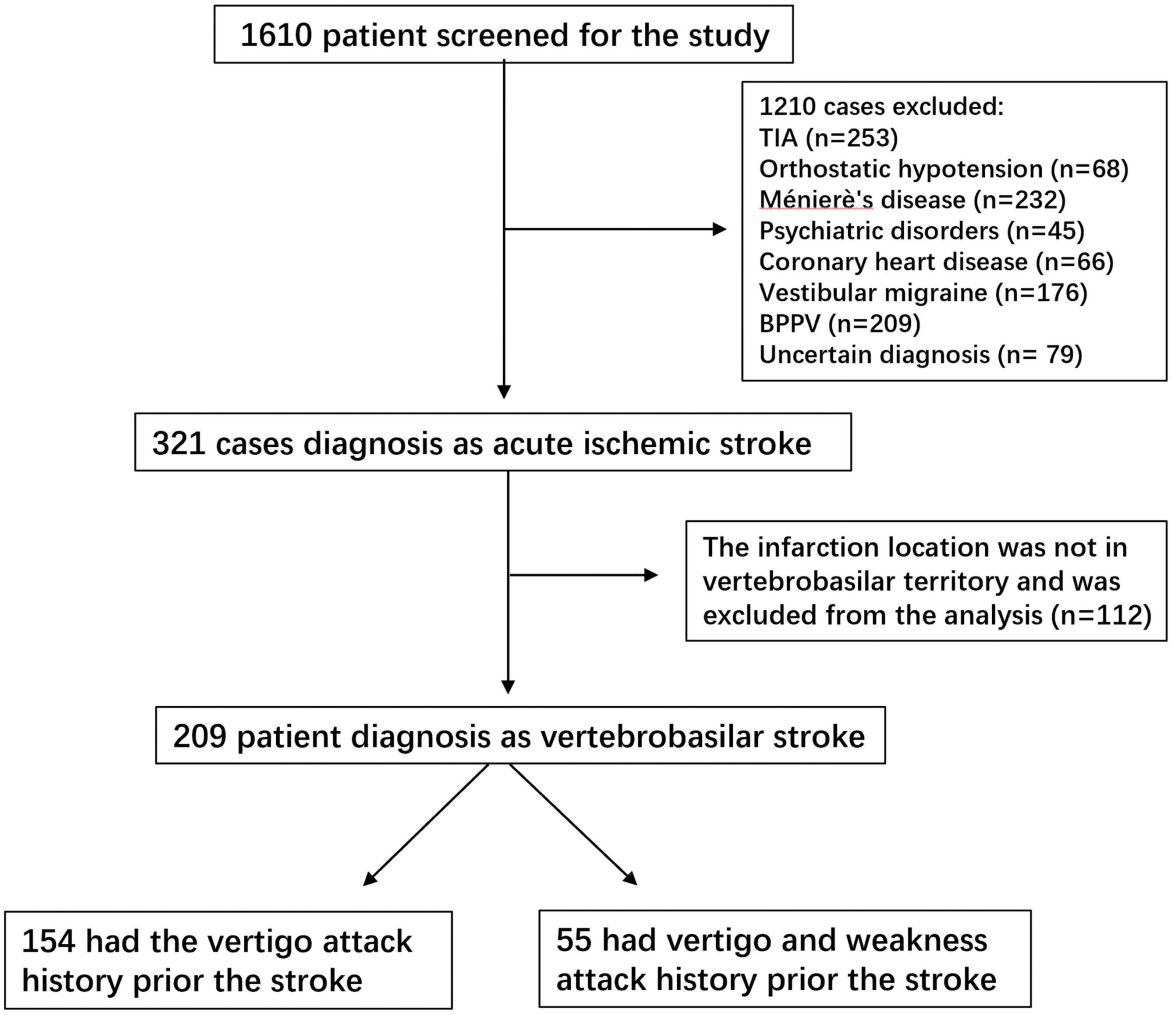
Study flow chart, TIA, and transient ischemic attack.

**Figure 2:**
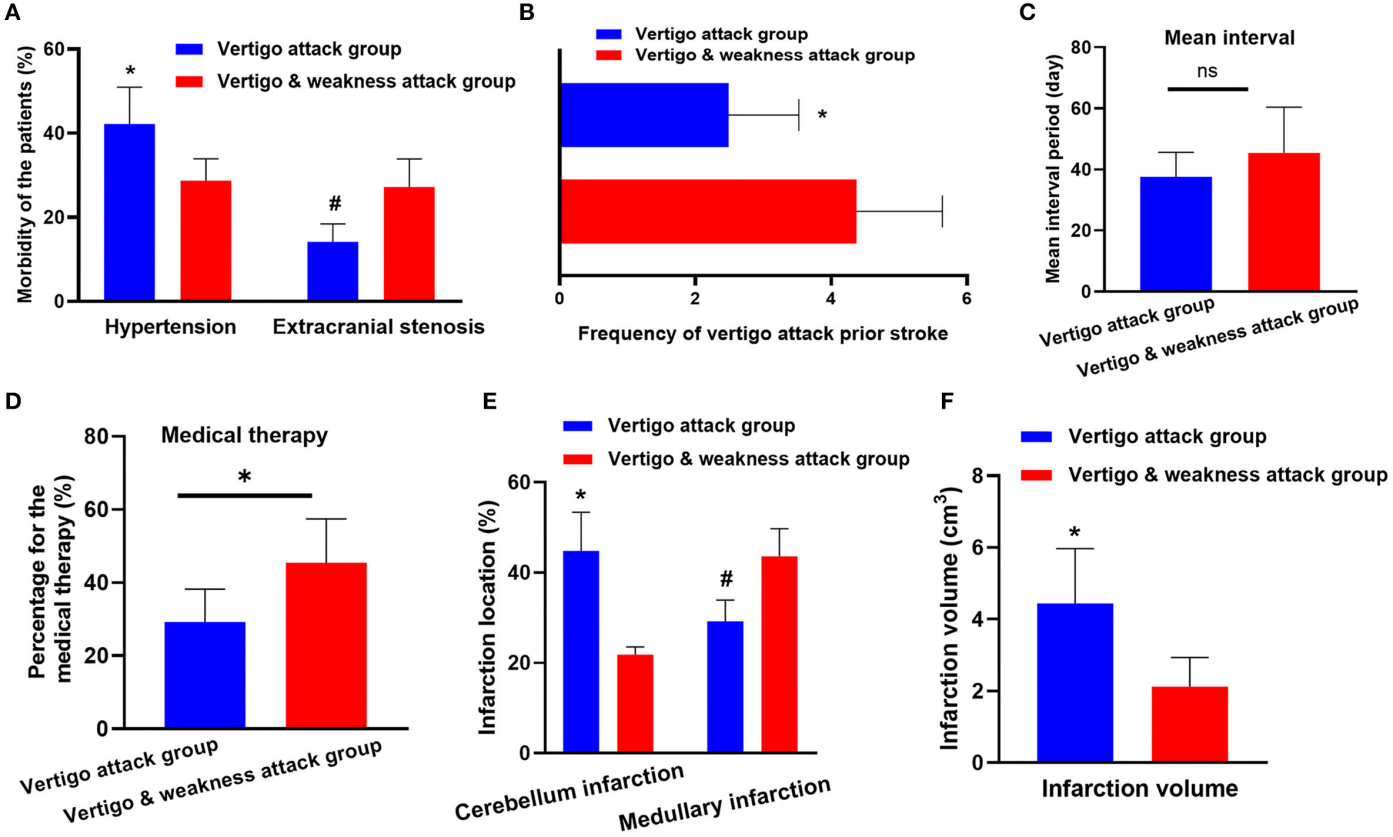
Clinical parameter comparison between the vertebrobasilar stroke (VO) group and the vertigo and weakness attacks (VW group). **(A)** The prevalence of hypertension is increased in the VO group (42.2 vs. 29.0%, *p* < 0.05). However, patients in the VW group had higher extracranial stenosis (14.2 vs. 27.2%, *p* < 0.05). **(B)** The frequency of vertigo attacks is higher in the VW group (median 2.4 vs. 4.3, *p* < 0.04). **(C)** The mean interval (the period from the onset of the vertigo attack to the diagnosis of ischemic stroke) shows no differences between the VO group and the VW group (median 37.6 vs. 45.4 days, *p* = 0.08). **(D)** Patients in the VW group are more likely to seek the medical attention after the vertigo symptom occurred (29.2 vs. 45.4%, *p* < 0.03). **(E)** The cerebellum is more frequently inflicted in the VO group (44.1 vs. 21.8%, *p* < 0.001). However, the medullary is more likely to be inflicted in the VW group (29.2 vs. 43.6%; *p* = 0.02). **(F)** The infarction volume in VO group is larger than the VW group (4.44 vs. 2.12 cm^3^, *p* < 0.05). * and # represents *p* < 0.05, ns, no statistical difference.

Patients hospitalized with vertigo symptoms as the main complaint were screened for this study (*n* = 1,610). Patients who were diagnosed with transient ischemic attack (*n* = 253), Ménierè's disease (*n* = 232), vestibular migraine (*n* = 176), Benign Paroxysmal Positional Vertigo (BPPV) (*n* = 209), vestibular neuritis (*n* = 107), orthostatic hypotension (*n* = 68), psychiatric disorders (*n* = 45), labyrinthitis (*n* = 54), and coronary heart disease (*n* = 66) were excluded from the analysis. Patients who did not receive a definite diagnosis were also excluded from the study (*n* = 79). Finally, 321 (19.9%) patients were definitively diagnosed with acute stroke. Among these stroke patients, 209 (65.1%) were in the vertebrobasilar territory, of which 154 had a vertigo attack history prior to vertebrobasilar stroke (VO group) and 55 had vertigo and weakness attacks (VW group). The vertigo duration in these patients ranged from 1 min to 24 h, and was mostly relieved within 30 min. The detailed information is presented in Figure 1. Vascular risk factors were compared between the two groups. Patients in the VW group had higher extracranial stenosis (14.2 vs. 27.2%, *p* < 0.05) and ABCD^2^ score (2.1 ± 1.2 vs. 3.6 ± 1.5, *p* = 0.02). However, the prevalence of hypertension was higher in the VO group (42.2 vs. 29.0%, *p* < 0.05; Figure 2A). Other vascular risk factors, such as diabetes mellitus (32.4 vs. 25.4%), hyperlipidemia (29.2 vs. 32.7%), alcohol consumption (18.8 vs. 21.8%), peripheral vascular disease (16.2 vs. 14.5%), and current smoking (25.3 vs. 30.9%) did not differ between the two groups (Table 1).

**Table 1:** Patient demographics and baseline characteristics (*n* = 209).

	**Vertigo attack group (*n* = 154)**	**Vertigo and weakness attack group (*n* = 55)**	***P*-value**
Male (n, %)	75 (48.7%)	30 (54.5%)	0.38
Age, Mean ± SD (years)	62.1 ± 11.2	59.8 ± 9.5	0.21
Hypertension (n, %)	65 (42.2%)	16 (29.0%)	<0.05^*^
Diabetes mellitus (n, %)	50 (32.4%)	14 (25.4%)	0.08
Body mass index, kg/m^2^	27.5 ± 5.2	26.3 ± 5.7	0.11
Coronary heart disease (n, %)	38 (24.6%)	15 (27.2%)	0.18
Extracranial stenosis (n, %)	22 (14.2%)	15 (27.2%)	<0.05^*^
Dyslipidemia (n, %)	45 (29.2%)	18 (32.7%)	0.32
Current smoking (n, %)	39 (25.3%)	17 (30.9%)	0.29
Alcoholism (n, %)	29 (18.8%)	12 (21.8%)	0.07
ABCD^2^ score, Mean ± SD	2.1 ± 1.2	3.6 ± 1.5	0.02^*^
Mean interval period (day)	37.6	45.4	0.08

* *Statistically significant*.

The frequencies of vertigo episodes before stroke were compared. As shown in Figure 2B, the frequency of vertigo attacks in the VW group was higher than in the VO group (median 2.4 vs. 4.3, *p* < 0.04). Additionally, although the mean interval (the period from the onset of vertigo attack to the ischemic stroke diagnosis) (median 37.6 vs. 45.4 days, *p* = 0.08) showed no differences between the two groups (Figure 2C), we found that patients in the VW group were more likely to seek medical attention after the vertigo symptom occurred (29.2 vs. 45.4%, *p* < 0.03; Figure 2D).

**Figure 3:**
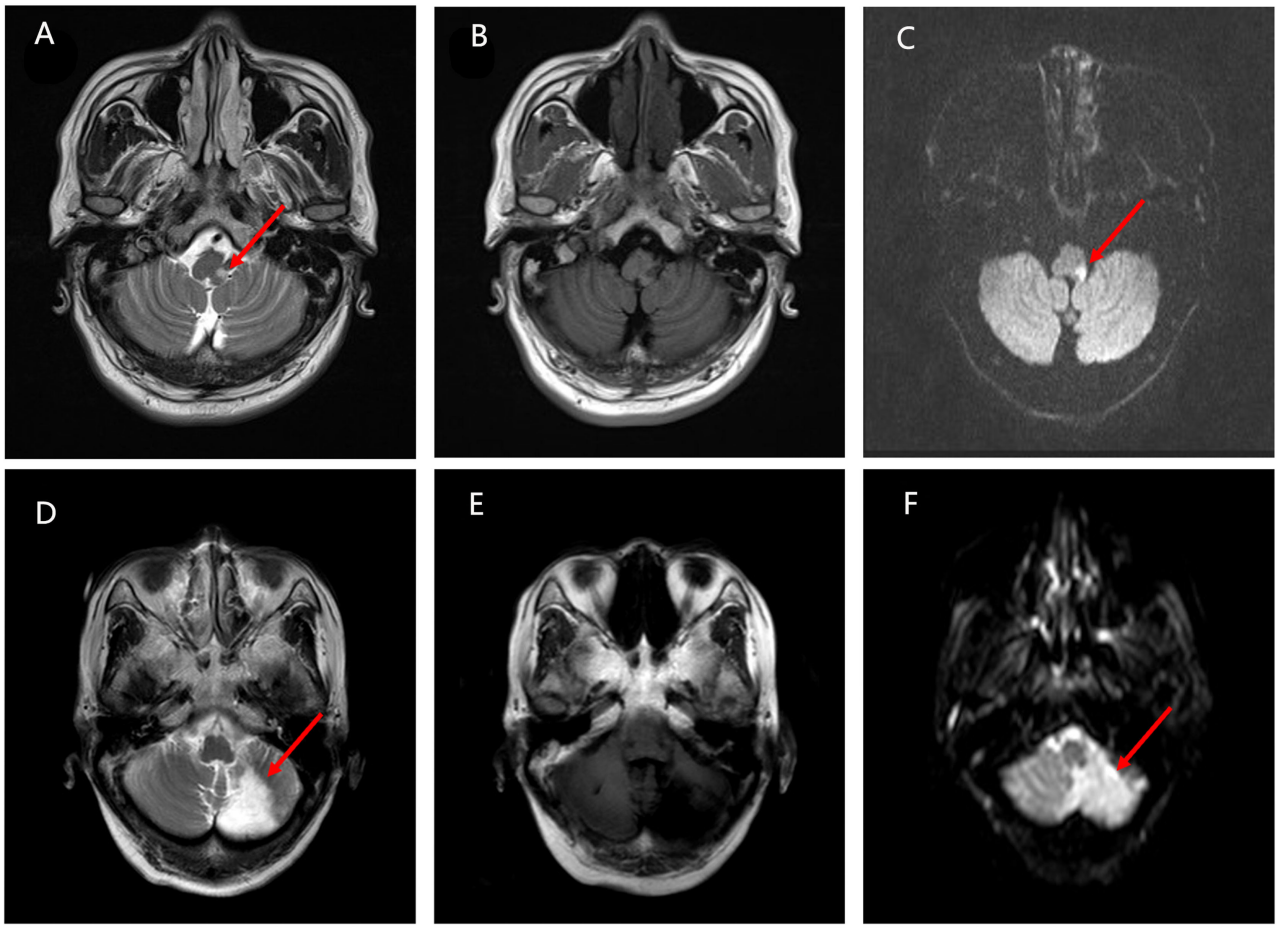
The differences in the infarction location between the VO group and the VW group. **(A–C)** Magnetic resonance imaging (MRI) from the VW group. T2 sequence axial MRI displays a tiny amount of increased signal located in the right side of the lateral medullary pointed by the red arrow. Diffusion-weighted imaging (DWI) sequences confirm the ischemic event. **(D–F)** MRI images from the VO group. MRI scan indicates an acute infarct in the cerebellum pointed by the red arrow. T2 sequence axial MRI displays a hyperintense area located in the left side of cerebellum. The ischemic event is confirmed by the DWI sequences further.

The location of the infarction was further evaluated between the groups. Notably, the cerebellum was more frequently inflicted in the VO group (44.1 vs. 21.8%, *p* < 0.001). However, the medulla was more likely to be inflicted in the VW group (29.2 vs. 43.6%; *p* = 0.02; Figure 2E). In addition, the infarction volume in the VO group was larger than the VW group (4.44 vs. 2.12 cm^3^, *p* < 0.05; Figures 2F, 3). A receiver operating characteristic curve was constructed to determine the relationship between the VO and VW groups in terms of infarction volume. A cut-off volume of >3.99 cm^3^ in cerebellar infarction was identified as an indicator of vertigo attack (specificity of 76.2%, sensitivity of 73.4%; *AUC* = 0.718 [95% confidence interval (*CI*), 0.615–0.820], and Youden index = 0.496, *p* = 0.002). The major findings of this study are summarized in supplementary 1.

## Discussion

The main causes of vertigo are the “peripheral” causes, such as Ménierè's disease and vestibular migraine. However, recurrent vertigo attacks could be an important focal neurological sign prior to an acute vertebrobasilar ischemic stroke. Notably, vertigo attacks preceding vertebrobasilar stroke frequently accompany other focal neurological deficits (19–21). Among these neurological symptoms, vertigo symptoms with weakness attacks on the face, arm, or soft palate are commonly observed prior to the vertebrobasilar stroke. However, the clinical features of vertigo with weakness attacks remain unclear. A total of 1,610 consecutive patients hospitalized with vertigo as the main complaint were screened for this study. Among them, 209 had a final diagnosis of vertebrobasilar stroke. Of these vertebrobasilar stroke cases, 154 had a vertigo attack history prior to stroke (VO group), and 55 had vertigo symptoms with a history of weakness attack (VW group). Those results suggested that patient who experienced vertigo attack only were more commonly occurred than vertigo accompanying weakness attack prior the acute vertebrobasilar stroke. By comparing the medical records of the two groups, we found that clinical parameters, such as infarction site, relative risk factors, and ABCD^2^ score, were different between the groups. These findings highlight the clinical features of patients with vertigo attacks only and those with vertigo accompanying weakness attacks prior to the vertebrobasilar stroke.

Neurodeficits associated with vertigo attacks before stroke are mainly dependent on the affected vascular territories (22, 23). However, the situation can be more complex and highly variable when vertigo attacks are accompanied by atypical neurological deficits. By comparing the clinical parameters between the VW and VO groups, we found that the frequency of vertigo attack episodes tended to be higher in the VW group. Additionally, the cerebellum was more likely to be affected in the VO group. The previous studies have shown that the cerebellum is mainly perfused by the posterior-inferior cerebellar artery (PICA) (22, 24), and vertigo attack is one of the most common signs of hypoperfusion in the cerebellum. Thus, it is reasonable to accept that vertigo attack was the main symptom observed when occlusion was inflicted in the Posterior-inferior cerebellar artery (PICA). In contrast, we demonstrated that the medullary region had a higher risk of being affected in the VW group. As the medulla is located in the middle of the brain stem, stenosis of the basilar artery dramatically decreases the perfusion of the medulla, contributing to vertigo and weakness attacks in these patients. Apart from vertigo and weakness, other neurological deficits, such as unilateral numbness and nausea, can also occur when hypoperfusion is afflicted in the medulla (15, 25). Based on these results, we conclude that the differences between the VO and VW groups were partly due to the artery in which the stenosis was afflicted.

Vertigo attacks provide an opportunity to prevent subsequent acute strokes (26, 27). The relative risk of stroke is as high as 60% in the following 3 months without timely intervention (28, 29). According to our data, the mean interval between the two groups was almost the same. Regrettably, fewer patients in the VO group sought medical intervention after the onset of vertigo symptoms. It is possible that patients who experienced both vertigo and weakness attacks experienced more discomfort than patients who only suffered a vertigo attack, which prompted them to make an urgent clinical appointment. Moreover, the prevalence of extracranial stenosis in the VW group was higher than that in the VO group, which reminded them to consult a primary physician regarding the most appropriate therapy. Stroke is the leading cause of severe disability. However, immediate clinical diagnosis following prompt medical intervention reduces the risk of subsequent stroke by up to 70% (30, 31). Thus, ascribing vertigo/vertigo accompanying weakness attacks as the vascular origin and prompt medical therapy are critical to reduce the stroke burden, especially in patients with diabetes, hypertension, or hyperlipidemia.

In summary, our findings highlight the clinical value of vertigo/vertigo accompanying weakness attacks before the vertebrobasilar stroke. However, this study has some limitations. First, patients with mild stroke were unwilling to visit the hospital if the neurological deficits resolved spontaneously. Therefore, the proportion of vertebrobasilar strokes may have been underestimated in this study. Second, the recruitment of patients in the study required large randomized multicenter clinical experiments. Third, this retrospective study depends on a review of historical records that were originally not designed to collect data for research. Thus, the collection of clinical features, such as the frequency and duration of vertigo, may have led to recall bias. It should also be acknowledged that the relatively small sample size may have resulted in selection bias.

## Conclusions

This study clarified the clinical features of patients with vertigo symptoms with or without a weakness attack prior to the vertebrobasilar stroke. Patients in the VO group generally had a higher prevalence of hypertension. The total cerebral infarction volume in this group was greater than that in the VW group. The infarcted territory in the VO group was more likely to be located in the cerebellum. The patients in the VW group had higher extracranial stenosis and ABCD^2^ scores. The percentage of patients with medullary infarction was higher in this group. Moreover, the frequency of vertigo attacks increased in the vertigo and weakness group. We also found that patients in the VW group were more willing to receive medical intervention before the occurrence of stroke. These findings highlight the different clinical features of patients with vertigo attack only and those with vertigo accompanying weakness attacks prior to vertebrobasilar ischemic stroke.

## Data Availability Statement

The original contributions presented in the study are included in the article/supplementary material, further inquiries can be directed to the corresponding authors.

## Ethics Statement

The studies involving human participants were reviewed and approved by Institutional Animal Ethics Committee of Xiangya Hospital. The patients/participants provided their written informed consent to participate in this study. All patients were recruited following informed consent.

## Author Contributions

YD drafted the manuscript. DQ and JH designed the study, provided the statistical analysis, and supervised the study. RZ, LZ, JH, and JD critically revised the manuscript for intellectual content. YD, JH, and DQ made the final revision of the manuscript. All authors have contributed to the manuscript and approved the submitted version.

## Funding

This study was supported by the National Science Foundation of China (No. 81974223) and Key Research and Development Program in Hunan Province (No. 2020SK2069).

## Conflict of Interest

The authors declare that the research was conducted in the absence of any commercial or financial relationships that could be construed as a potential conflict of interest.

## Publisher's Note

All claims expressed in this article are solely those of the authors and do not necessarily represent those of their affiliated organizations, or those of the publisher, the editors and the reviewers. Any product that may be evaluated in this article, or claim that may be made by its manufacturer, is not guaranteed or endorsed by the publisher.
